# Supplementary material for: A nonlinear updating algorithm captures suboptimal inference in the presence of signal-dependent noise
Source: Sci Rep. 2018 Aug 22;8:12597. doi: 10.1038/s41598-018-30722-0 (PMC6105733; doi:10.1038/s41598-018-30722-0)
Supplement: Supplementary file 1 — Supplementary Information [file 41598_2018_30722_MOESM1_ESM.pdf]

A nonlinear updating algorithm captures suboptimal inference in the presence of signal-dependent noise

Seth W. Egger and Mehrdad Jazayeri

Supporting information

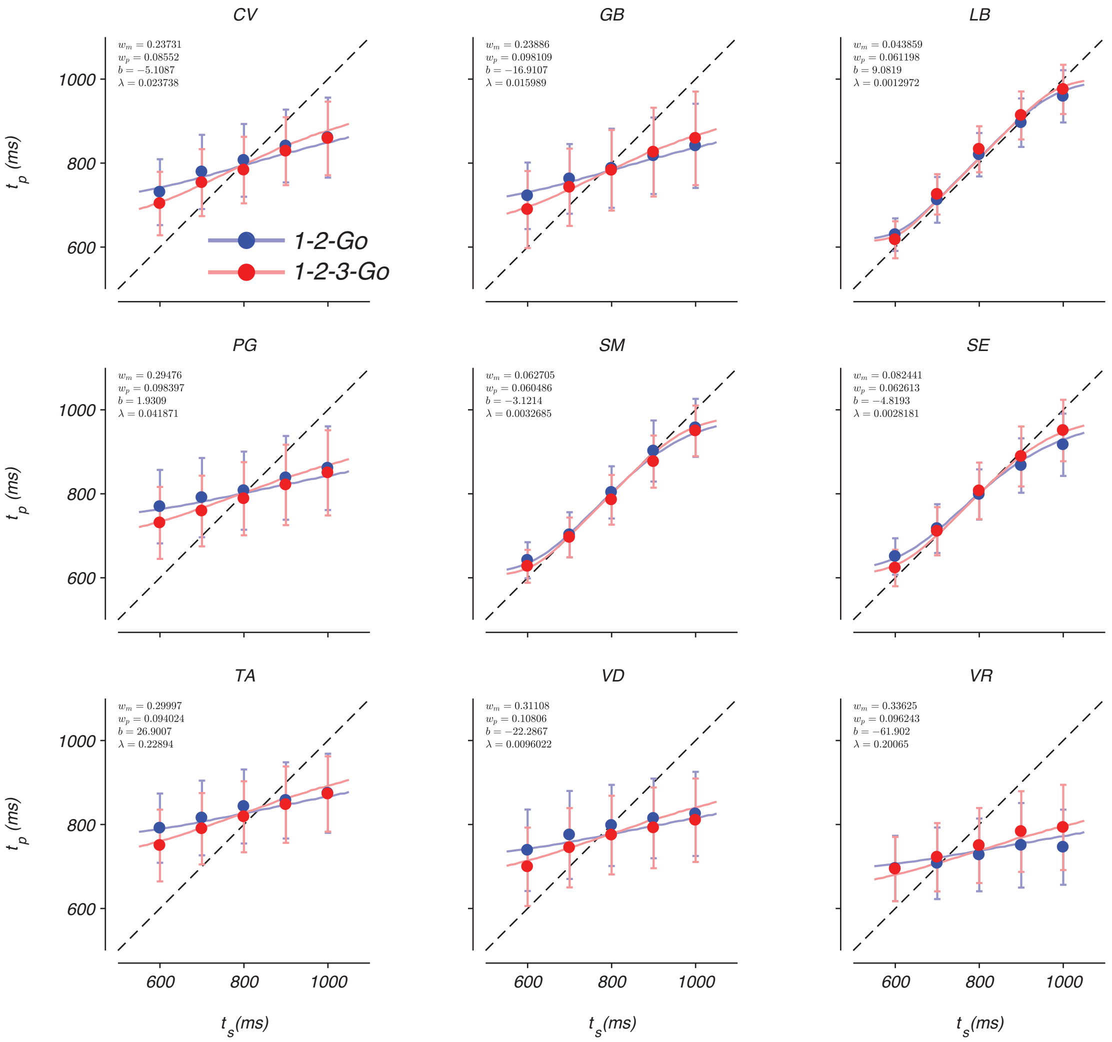

Supplementary Figure 1. Fit of the BLS model to each subject. Mean and standard deviation (circles and error bars) of the produced interval, t_p, as a function of the sample interval, t_s, for each subject in 1-2-Go (blue) and 1-2-3-Go (red) trials. The dashed line indicates unity and the solid lines depict the BLS model for each subject. Insets: maximum likelihood estimate of model parameters for each subject.

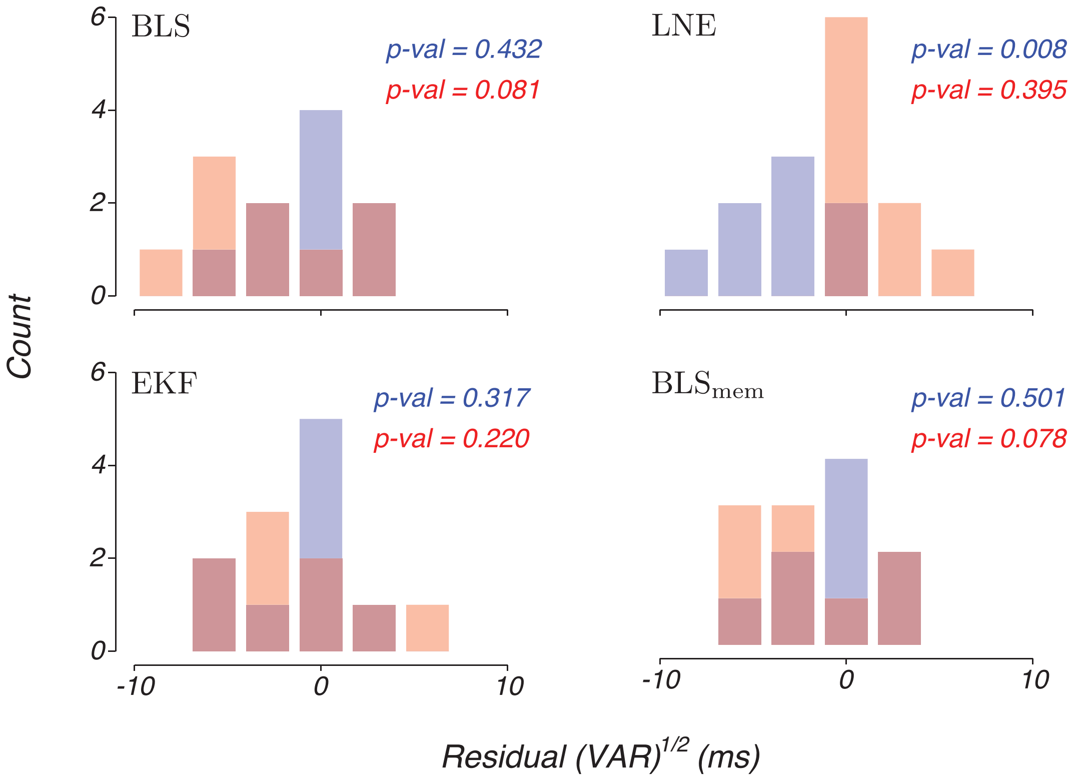

Supplementary Figure 2. Residual $\sqrt{\text{VAR}}$ compared to compared to prediction of each model. Difference between the observed and predicted $\sqrt{\text{VAR}}$ for 1-2-Go (blue) and 1-2-3-Go (red) for each of the candidate models. Insets indicate the results of a t-test (d.f. = 8) across subjects testing for a difference between model and behavior. The LNE model systematically overpredicts the variance observed in the 1-2-Go condition. No other systematic deviations were detected.

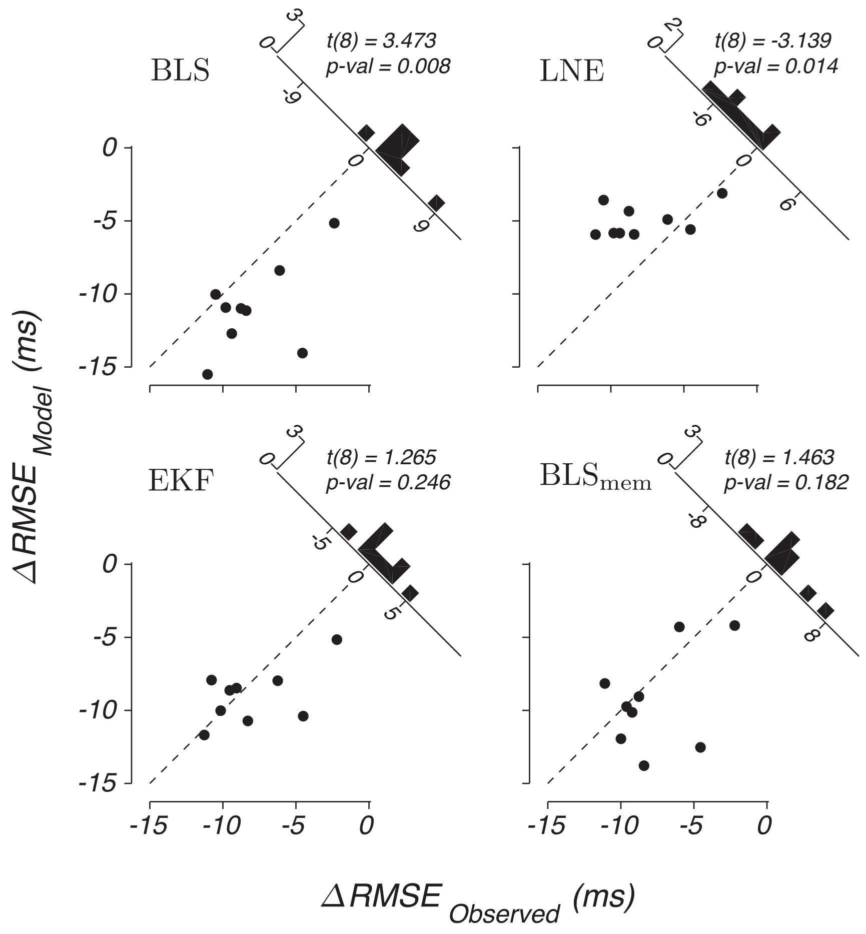

Supplementary Figure 3. Residual difference in RMSE between 1-2-Go to 1-2-3-Go compared to prediction of each model. The scatter plots show the change in RMSE predicted by a fit model (ordinate) to change in RMSE in subjects' data (abscissa). Each point corresponds to one subject. Histograms show the difference between model and data. The result of a t-test indicated the BLS model systematically underestimated the change in RMSE compared to the data. LNE, on the other hand, overestimated the difference in RMSE. The difference between model predictions and data was not significant for the EKF and BLS_{mem} models.

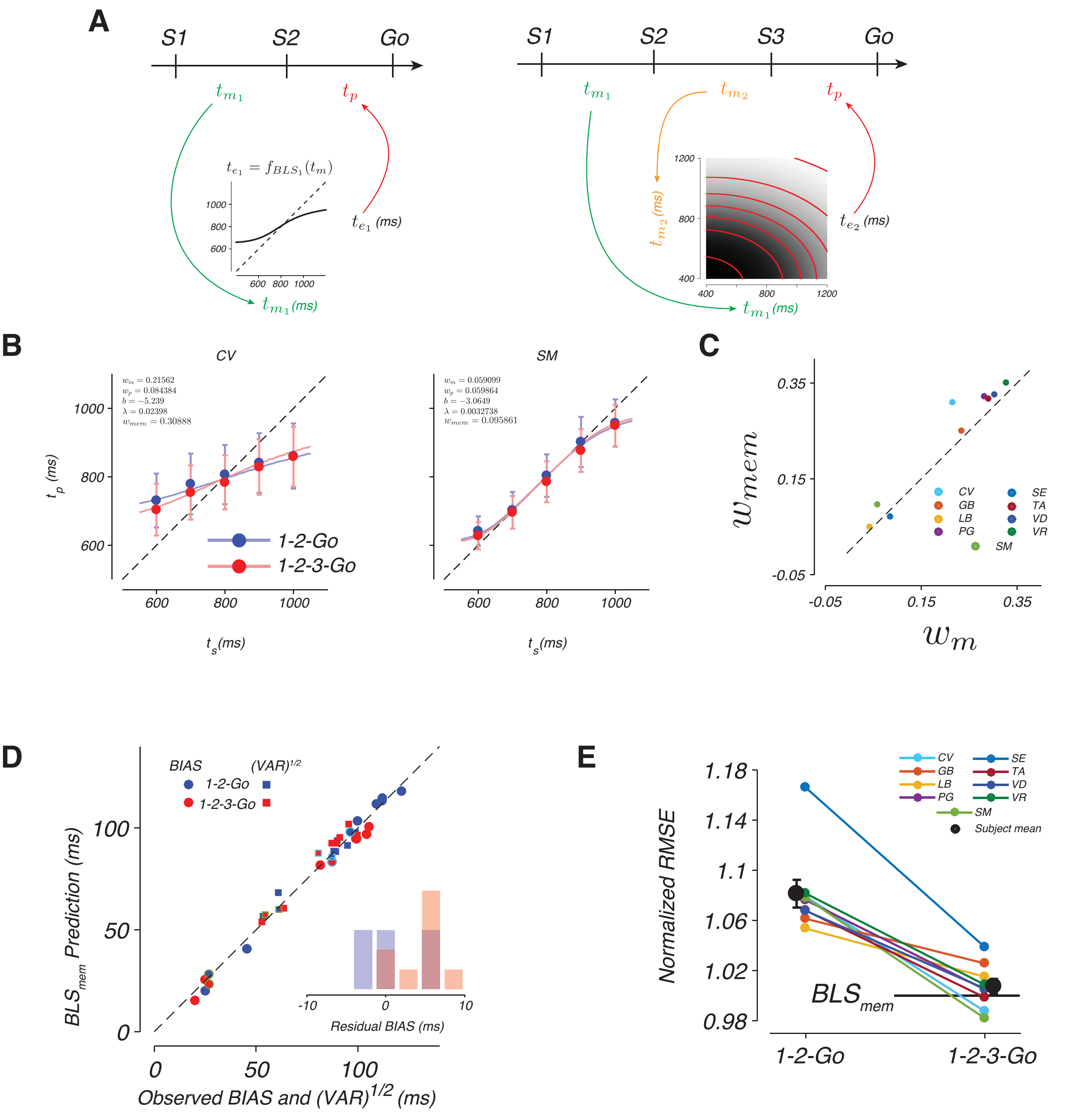

Supplementary Figure 4. **BLS with different levels of noise for the two measurements ($\mathbf{BLS_{mem}}$).** (A) Left: For the 1-2-Go condition, BLS_{mem} is identical to BLS. Right: For the 1-2-3-Go condition, BLS_{mem} assumes that the Weber fraction for the measurement immediately before production, w_m is the same as the 1-2-Go condition, but the Weber fraction of the first measurement, denoted by w_{mem}, could be different from w_m. Therefore, the BLS_{mem} model has one more parameter than the BLS model. The bottom grayscale shows the estimate, t_{e_2}, as a function of t_{m_1} and t_{m_2} for $w_{mem} = 0.225$ and $w_m = 0.15$. Comparison of the iso-estimate contours (red lines) to the BLS mapping function (Figure 3D), indicates an increased reliance on t_{m_2}. (B) Predictions of the BLS_{mem} model (lines) and mean t_p +/- the standard deviation of example subjects (circles and error bars) as a function of t_s. Conventions are the same as panels shown in Supplementary Figure 1. (C) Maximum likelihood w_{mem} plot against w_m fit to each subject. Dashed line plots unity. In only one subject w_m exceeded w_{mem}. (D) Subject BIAS and $\sqrt{\text{VAR}}$ plotted against the predictions of the BLS_{mem} model fit to each subject. Inset: difference between the expected BIAS based on the BLS_{mem} model and that observed. (E) Each subject's RMSE in the 1-2-Go and 1-2-3-Go conditions to normalized by RMSE expected from the BLS_{mem} model in the 1-2-3-Go condition. See also Table 1.

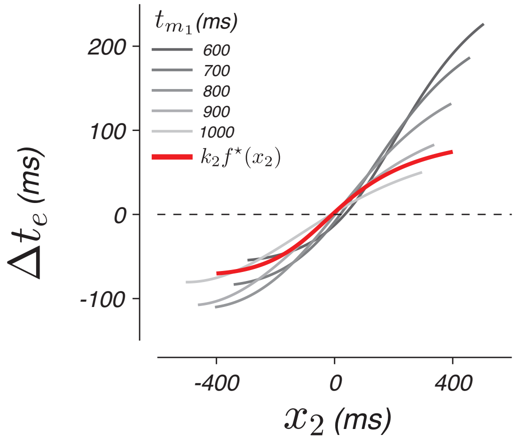

Supplementary Figure 5. The update function which achieves the BLS estimate after two measurements. The BLS estimator for the 1-2-3-Go task uses the mean of the posterior, t_{e_2} after updating the posterior with the likelihood function associated with the second measurement, t_{m_2}. We characterized the function of the BLS estimator in terms of a linear updating term Δt_e. This term quantified the magnitude by which t_{e_1} has to be updated after the second measurement. A plot of Δt_e as a function of the error between the first estimate and the second measurement ($x_2 = t_{m_2} - t_{e_1}$) reveals two notables features. First the updating is a nonlinear function of x_2, and second, the form of the nonlinearity depends on t_{m_1} (different grayscales). In other words, BLS can be implemented with a linear updating scheme based on errors only if the update is derived from a nonlinear function whose form of nonlinearity varies with t_{m_1}. The red curve corresponds to the nonlinear updating scheme implemented by the EKF model, $k_2 f^\star(x_2)$. In this case, the updating relies on a nonlinear function of error but the form of the nonlinearity does not vary with t_{m_1}. See also “An algorithmic view of Bayesian integration” and “An extended Kalman filter (EKF) model for approximate Bayesian inference”.

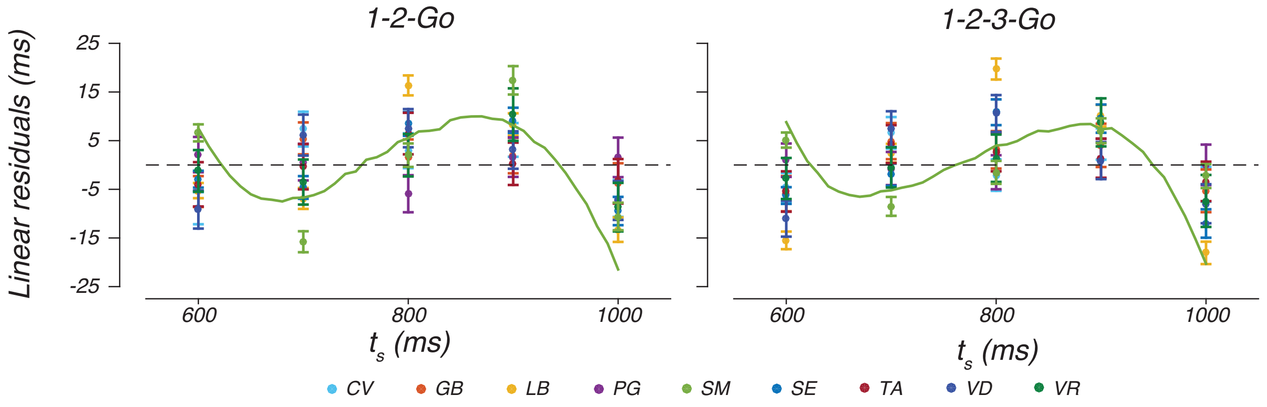

Supplementary Figure 6. Nonlinear processing of intervals. Mean residuals of the best fitting linear regression model ($\hat{t}_p = \beta t_s + \gamma$) relating production interval (t_p) to sample interval (t_s) for all subjects (different colors). Each data point represents the mean residual (e.g. $\frac{1}{M}\sum_{i=1}^{M}(t_{p_i} - \hat{t}_p)$, with i indexing trials). The error bars represent the standard error of the residuals. Residuals should be near zero (dashed line) if t_p was linearly related to t_s. The solid green line depicts the expected residuals from the BLS model fits to data from one subject (SM). The predictions for other subjects are qualitatively similar, but the exact shape depends on the measurement noise for each subject. The deviation of the data from the linear model indicates that subjects utilized a nonlinear transfer function to estimate t_s. See also Section "A linear-nonlinear estimator (LNE) model for approximate Bayesian inference".

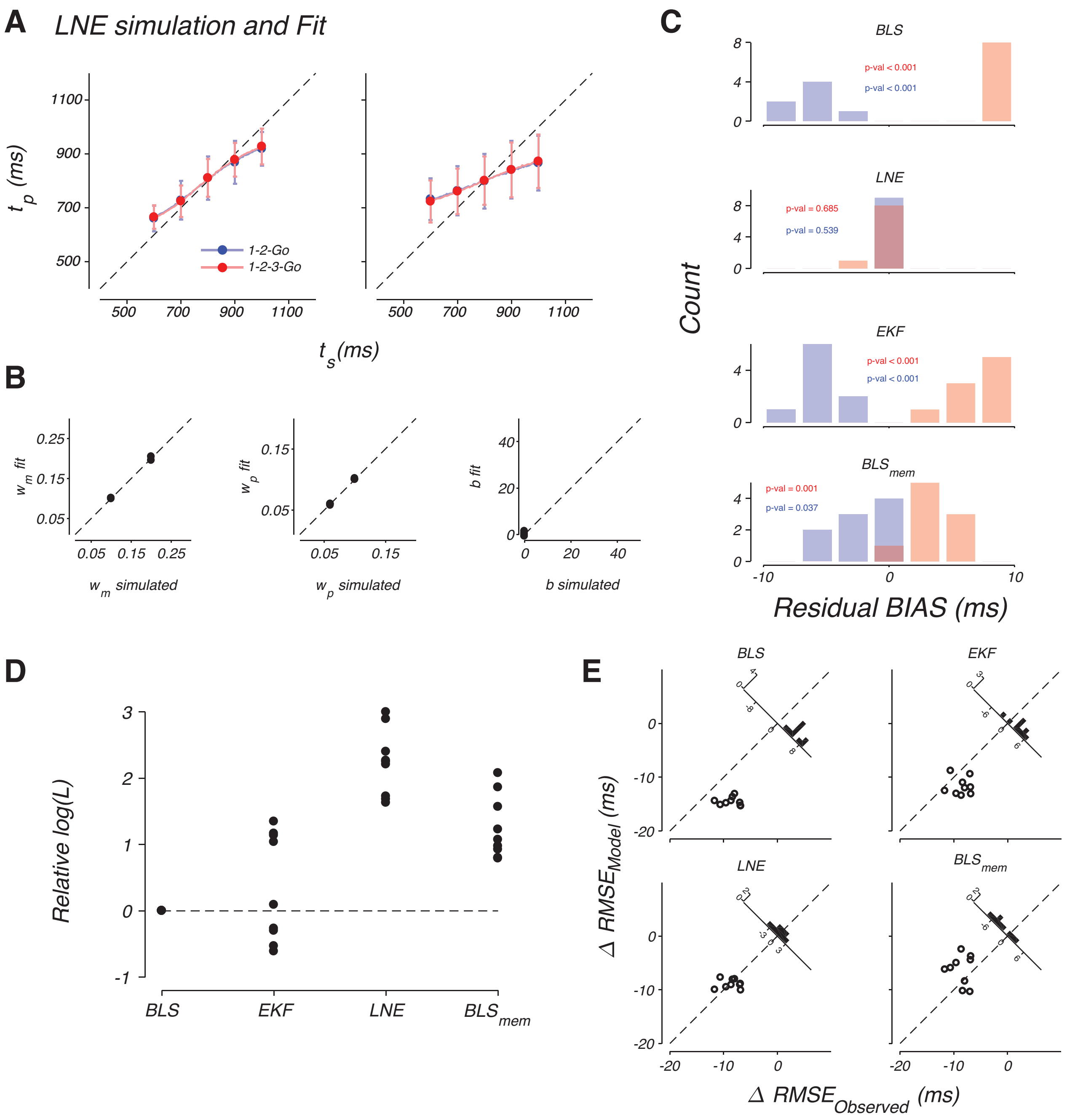

Supplementary Figure 7. Simulation of behavior implementing the LNE algorithm and subsequent analysis. We simulated 9 observers whose behavior followed the generative process associated with LNE. Each observer performed 2500 trials of each condition (1-2-Go and 1-2-3-Go). Since our subject pool comprised of both low noise and high noise individuals, each LNE observer model was randomly assigned to be either low noise ($w_m = 0.1$ and $w_p = 0.06$, respectively) or a high noise ($w_m = 0.2$ and $w_p = 0.1$, respectively). (A) Interval reproduction behavior of two simulated LNE observers. Conventions as in Supplementary Figure 1. (B) Maximization of the log likelihood under the LNE model recovers the correct parameters. The left, middle, and right panels show fits to w_m, w_p, and b as a function of the corresponding parameters used in the generative model. (C) Residual BIAS for each model fit to the simulated data. Residual BIAS was smallest for the LNE model. The data was systematically more biased in 1-2-3-Go conditions and less biased in 1-2-Go conditions compared to the BLS fits. The same was true for the EKF model, but to a lesser extent. (D) Log likelihood of the data under each model, relative to the BLS model. The LNE model was most likely given the data. Results indicate that our model fitting is able to correctly identify a LNE observer compared to the other potential models. (E) Change in RMSE of the simulated data plot against the change in RMSE expected under each model. The LNE model correctly predict the change in RMSE while both the BLS and EKF

models overestimate the reduction. Finally the BLS_{mem} model underestimates the RMSE.

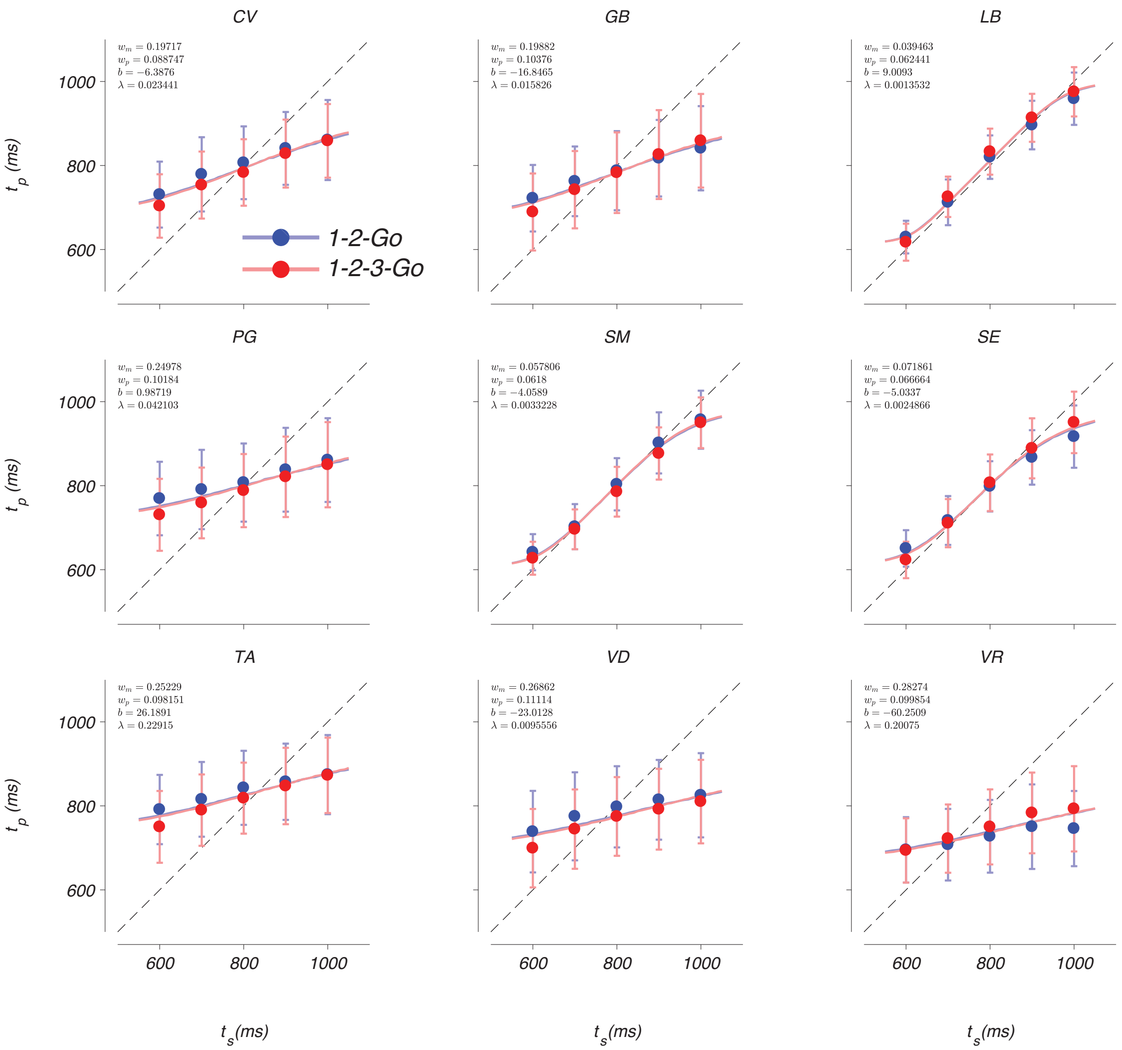

Supplementary Figure 8. **Fit of the LNE model to each subject.** Conventions are the same as Supplementary Figure 1 but for the LNE model.

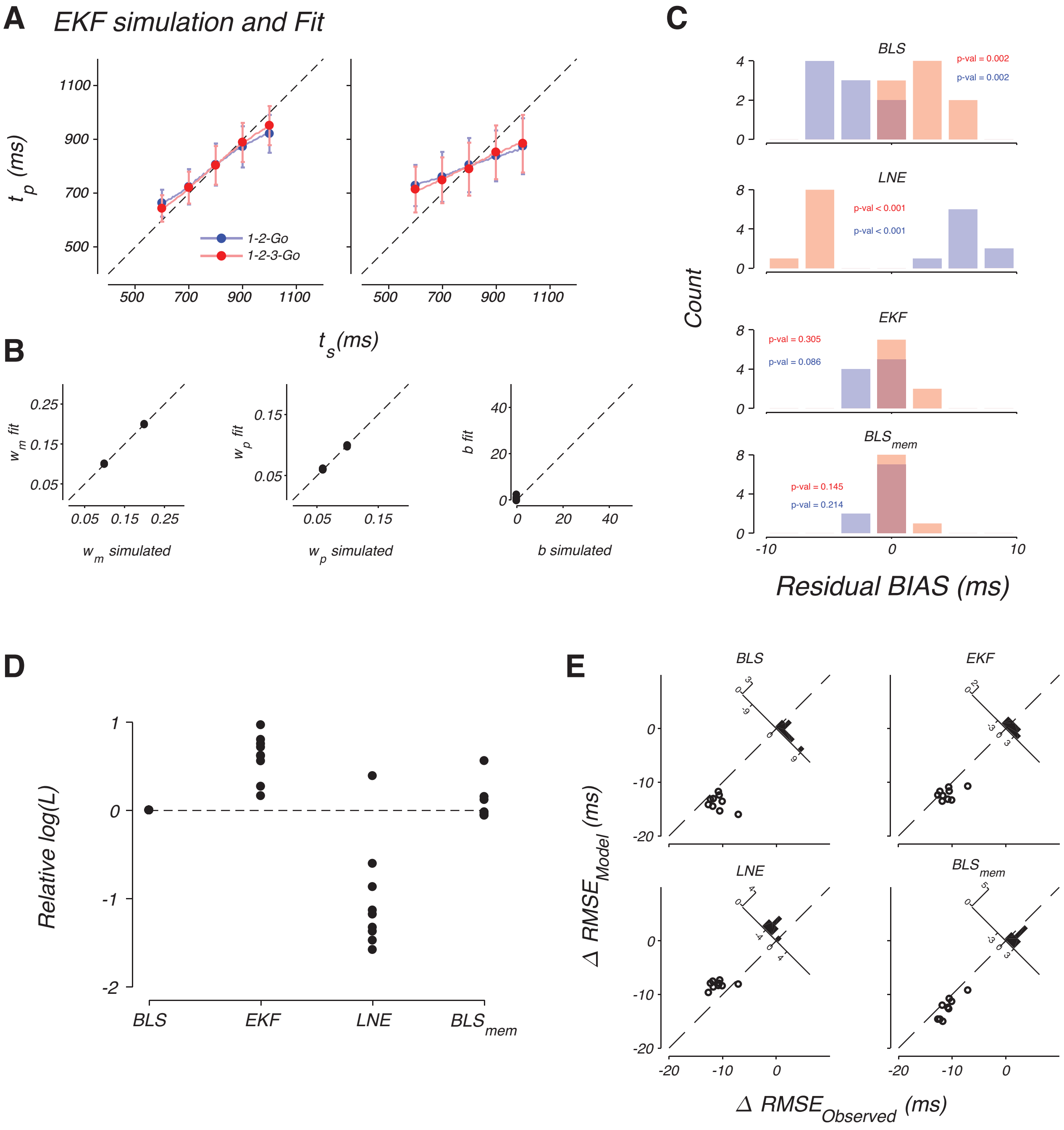

Supplementary Figure 9. Simulation of behavior implementing the EKF algorithm and subsequent analysis. We simulated 9 observers whose behavior followed the generative process associated with EKF. Each observer performed 2500 trials of each condition (1-2-Go and 1-2-3-Go). Since our subject pool comprised of both low noise and high noise individuals, each EKF observer model was randomly assigned to be either low noise ($w_m = 0.1$ and $w_p = 0.06$, respectively) or a high noise ($w_m = 0.2$ and $w_p = 0.1$, respectively). (A) Interval reproduction behavior of two simulated EKF observers. Conventions as in Supplementary Figure 7. (B) Maximization of the log likelihood under the EKF model recovers the correct parameters. The left, middle, and right panels show fits to w_m, w_p, and b as a function of the corresponding parameters used in the generative model. (C) Residual BIAS for each model fit to the simulated data. Residual BIAS was smallest for the EKF model. The data was systematically more biased in 1-2-3-Go conditions and less biased in 1-2-Go conditions compared to the BLS fits. In contrast, the data was systematically less biased in 1-2-3-Go and more biased in 1-2-Go when compared with the LNE model. (D) Log likelihood of the data under each model, relative to the BLS model. The EKF model was most likely given the data. Results indicate that our model fitting is able to correctly identify a EKF observer compared to the other potential models. (E) Change in RMSE of the simulated data plot against the change in RMSE expected under each model. The EKF model correctly

predicted the change in RMSE while both the BLS and BLS_{mem} models overestimate the reduction. Finally the LNE model underestimates the change in RMSE.

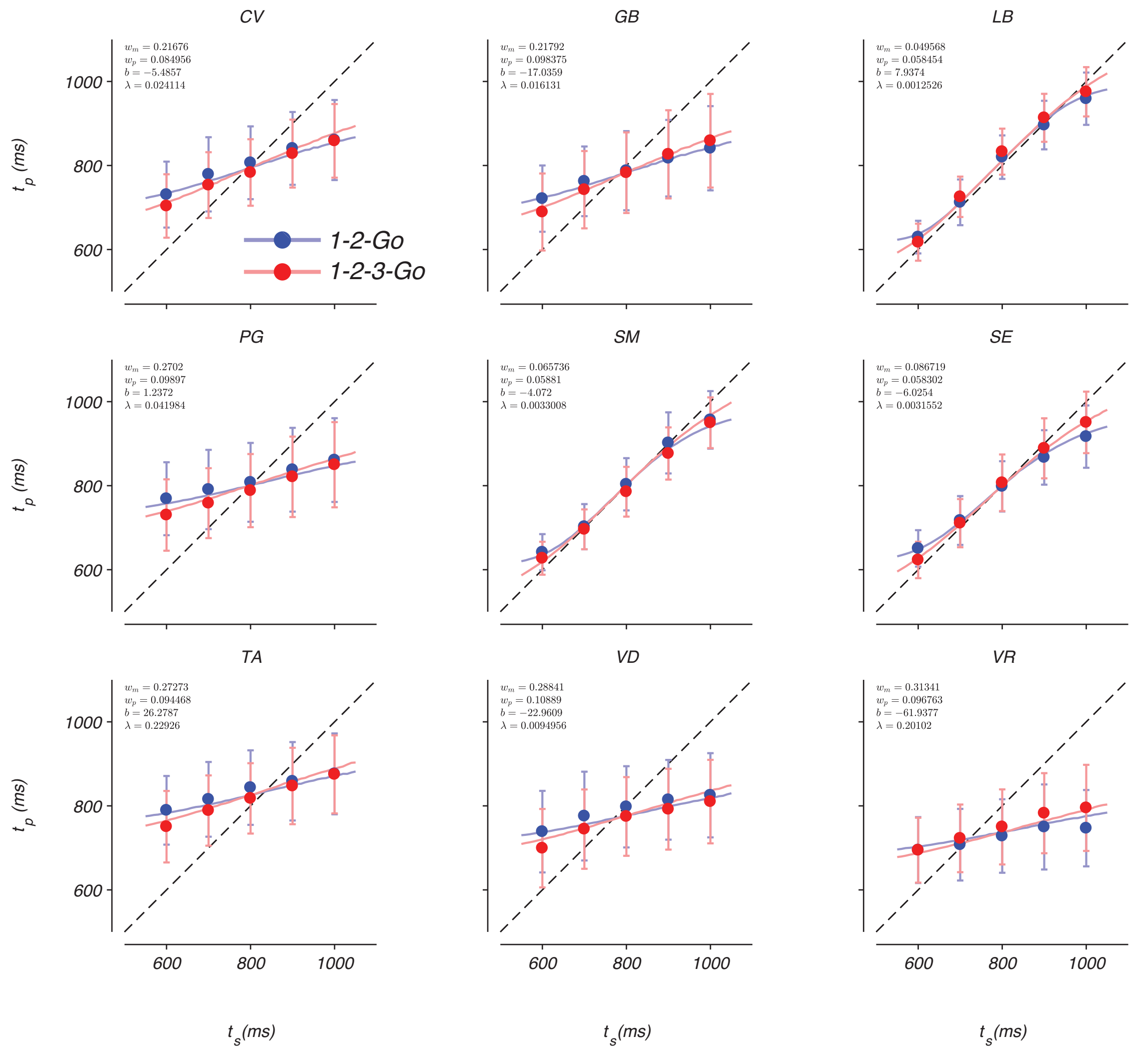

Supplementary Figure 10. Fit of the EKF model to each subject. Conventions are the same as Supplementary Figure 1 but for the EKF model.

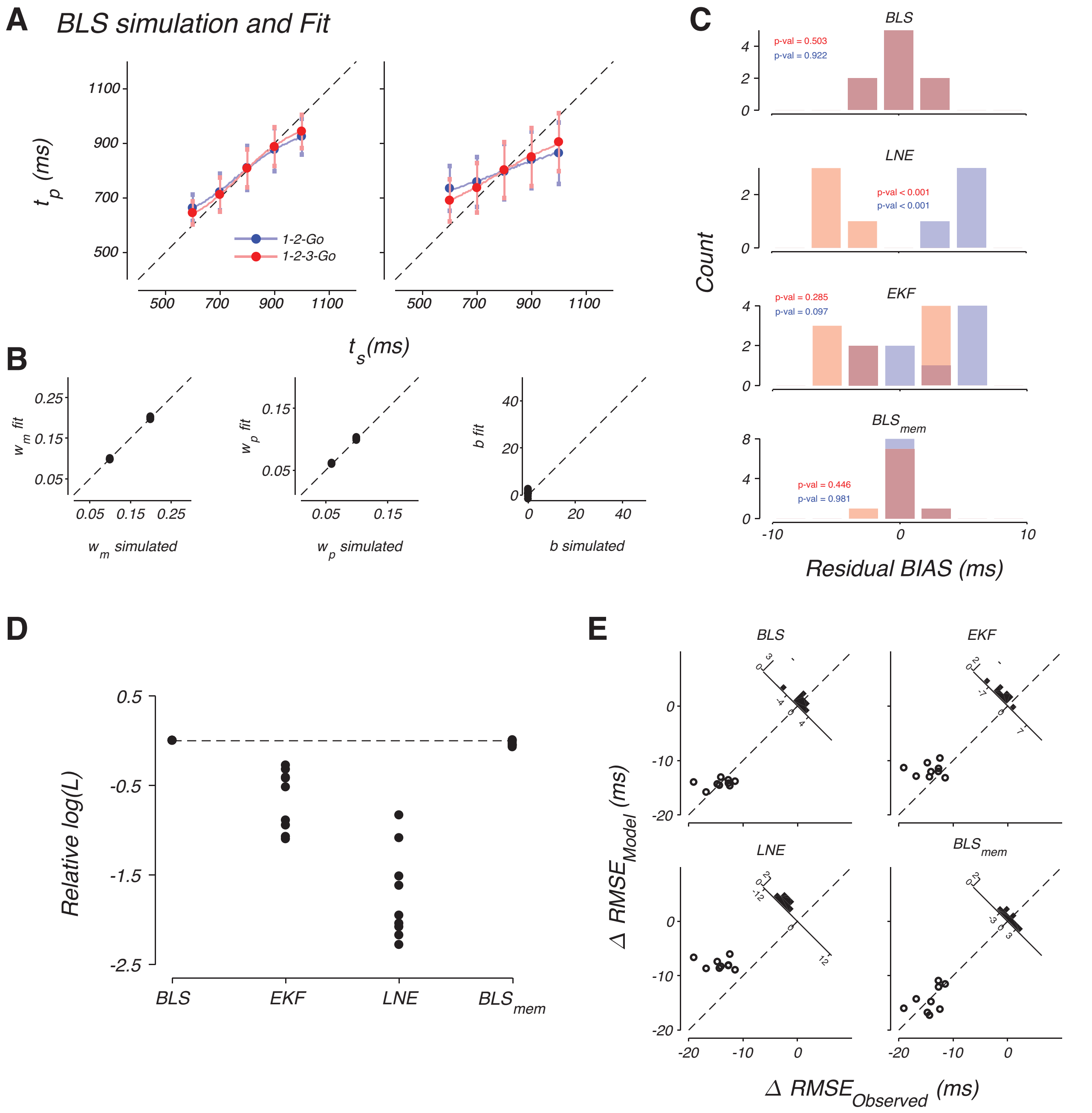

Supplementary Figure 11. Simulation of behavior implementing the BLS algorithm and subsequent analysis. We simulated 9 observers whose behavior followed the generative process associated with BLS. Each observer performed 2500 trials of each condition (1-2-Go and 1-2-3-Go). Since our subject pool comprised of both low noise and high noise individuals, each BLS observer model was randomly assigned to be either low noise ($w_m = 0.1$ and $w_p = 0.06$, respectively) or a high noise ($w_m = 0.2$ and $w_p = 0.1$, respectively). (A) Interval reproduction behavior of two simulated BLS observers. Conventions as in Supplementary Figure 7. (B) Maximization of the log likelihood under the BLS model recovers the correct parameters. The left, middle, and right panels show fits to w_m, w_p, and b as a function of the corresponding parameters used in the generative model. (C) Residual BIAS for each model fit to the simulated data. Residual BIAS was smallest for the BLS and BLS$_{\text{mem}}$ models. The data was systematically more biased in 1-2-Go conditions and less biased in 1-2-3-Go conditions compared to the LNE fits. The same was true for the EKF model, but to a lesser extent. (D) Log likelihood of the data under each model, relative to the BLS model. The BLS and BLS$_{\text{mem}}$ models were most likely given the data. Results indicate that our model fitting is able to correctly identify a BLS observer compared to LNE and EKF models. The BLS$_{\text{mem}}$ model is also consistent with the data which is expected because it is identical to the BLS model when w_{mem} equals w_m. (E) Change in RMSE of the simulated data plot against the change in RMSE expected under each model. The BLS and BLS$_{\text{mem}}$ models correctly identify

the change in RMSE while both the LNE and EKF models underestimate the reduction.

		Subject								
	Condition	CV	GB	LB	PG	SM	SE	TA	VD	VR
	1-2-Go	130.24	134.01	59.12	144.50	66.68	76.58	141.39	149.89	147.86
RMSE	1-2-3-Go	119.23	129.51	56.79	134.74	60.62	68.22	132.04	141.18	137.41

Supplementary Table 1. **RMSE by subject, trial type.** Summary of the RMSE in reproduction behavior by subject and trial type.

		Subject								
	Condition	CV	GB	LB	PG	SM	SE	TA	VD	VR
Total trials	1-2-Go (lapse)	3096 (68)	3156 (36)	3138 (5)	3130 (117)	3120 (10)	3143 (11)	2565 (587)	3099 (18)	2103 (371)
	1-2-3-Go (lapse)	3132 (38)	3120 (32)	3169 (1)	3084 (72)	3067 (5)	3159 (3)	2775 (415)	3182 (16)	2108 (316)

Supplementary Table 2. Trials completed by condition and subject in the interval reproduction task. Summary of the number of trials for the interval reproduction task in each condition and the number of trials identified as lapses in parentheses.

	Parameters		Subjects								
			CV	GB	LB	PG	SM	SE	TA	VD	VR
BLS	w_m	Full data	0.237	0.239	0.044	0.295	0.063	0.082	0.300	0.311	0.336
		LNOCV	0.237	0.239	0.044	0.295	0.063	0.082	0.300	0.311	0.336
	w_p	Full data	0.086	0.098	0.061	0.098	0.061	0.063	0.094	0.108	0.096
		LNOCV	0.086	0.098	0.061	0.098	0.061	0.063	0.094	0.108	0.096
	b	Full data	-5.10	-16.92	9.07	1.93	-3.12	-4.82	26.92	-22.29	-61.91
		LNOCV	-5.12	-16.92	9.08	1.93	-3.12	-4.82	26.91	-22.29	-61.90
	γ	Full data	0.024	0.016	0.001	0.042	0.003	0.003	0.229	0.010	0.201
		LNOCV	0.024	0.016	0.001	0.042	0.003	0.003	0.229	0.010	0.201
LNE	w_m	Full data	0.197	0.199	0.040	0.250	0.058	0.072	0.252	0.269	0.283
		LNOCV	0.197	0.199	0.040	0.250	0.058	0.072	0.252	0.269	0.283
	w_p	Full data	0.089	0.104	0.062	0.102	0.062	0.067	0.098	0.111	0.100
		LNOCV	0.089	0.104	0.062	0.102	0.062	0.067	0.098	0.111	0.100
	b	Full data	-6.38	-16.85	9.00	0.99	-4.05	-5.04	26.19	-23.02	-60.27
		LNOCV	-6.39	-16.85	9.01	0.99	-4.06	-5.03	26.19	-23.01	-60.25
	γ	Full data	0.023	0.016	0.001	0.042	0.003	0.003	0.229	0.010	0.201
		LNOCV	0.023	0.016	0.001	0.042	0.003	0.003	0.229	0.010	0.201
EKF	w_m	Full data	0.217	0.218	0.050	0.270	0.066	0.087	0.273	0.288	0.314
		LNOCV	0.217	0.218	0.050	0.270	0.066	0.087	0.273	0.288	0.313
	w_p	Full data	0.085	0.098	0.059	0.099	0.059	0.058	0.095	0.109	0.097
		LNOCV	0.085	0.098	0.059	0.099	0.059	0.058	0.095	0.109	0.097
	b	Full data	-5.48	-17.04	7.93	1.23	-4.07	-6.03	26.29	-22.96	-61.95
		LNOCV	-5.49	-17.04	7.94	1.24	-4.07	-6.03	26.29	-22.97	-61.96
	γ	Full data	0.024	0.016	0.001	0.042	0.003	0.003	0.229	0.010	0.201
		LNOCV	0.024	0.016	0.001	0.042	0.003	0.003	0.229	0.010	0.201
BLS_{mem}	w_m	Full data	0.216	0.234	0.043	0.282	0.059	0.086	0.291	0.303	0.328
		LNOCV	0.216	0.235	0.043	0.282	0.059	0.086	0.291	0.303	0.328
	w_p	Full data	0.084	0.098	0.061	0.098	0.060	0.063	0.094	0.108	0.096
		LNOCV	0.084	0.098	0.061	0.098	0.060	0.063	0.094	0.108	0.096
	b	Full data	-5.23	-16.89	8.97	1.91	-3.06	-4.73	26.85	-22.31	-61.80
		LNOCV	-5.24	-16.89	8.98	1.92	-3.07	-4.73	26.83	-22.31	-61.79
	γ	Full data	0.024	0.016	0.001	0.042	0.003	0.003	0.229	0.010	0.201
		LNOCV	0.024	0.016	0.001	0.042	0.003	0.003	0.229	0.010	0.201
	w_{mem}	Full data	0.309	0.250	0.049	0.321	0.095	0.071	0.316	0.325	0.350
		LNOCV	0.309	0.250	0.049	0.321	0.096	0.071	0.316	0.325	0.350

Supplementary Table 3. Maximum likelihood parameters found by fitting the full data set and LNOCV. Each entry to the table indicates the maximum likelihood parameter found by fitting the entire data set (Full data) or by maximizing the likelihood of left out data for cross validation (LNOCV; see Methods).

Appendix

We modeled the likelihood of a sample, s, after making a measurement, m_i, perturbed by scalar noise as follows:

$$p(m_i|s) = \frac{1}{\sqrt{2\pi w_m^2 s^2}} e^{-\frac{(m_i-s)^2}{2w_m^2 s^2}}, \tag{1}$$

where the parameter w_m scales the standard deviation of the noise with the magnitude of the signal. For N conditionally independent measurements with the same w_m, the likelihood can be written as follows:

$$p(m_1, m_2, ..., m_N|s) = \left(\frac{1}{\sqrt{2\pi w_m^2 s^2}}\right)^N e^{-\frac{\sum_{i=1}^{N}(m_i-s)^2}{2w_m^2 s^2}}, \tag{2}$$

In Eq (2), because the term in the parentheses, which depends on s, is exponentiated to the power of N, we can conclude that the form of the likelihood function is not invariant with respect to the number of measurements. Therefore, in the presence of scalar noise, to perform exact estimation, it is necessary to update the full distribution after each measurement.

The maximum likelihood estimator (MLE) associated with Eq (2) can be derived analytically by setting the derivative of the logarithm of the likelihood function to zero, which leads to the following solution:

$$f_{MLE}(m_1, m_2, ..., m_N) = \overline{m}\left[\frac{-1+\sqrt{1+4w_m^2\frac{\overline{m^2}}{\overline{m}^2}}}{2w_m^2}\right], \tag{3}$$

where

$$\overline{m} = \frac{1}{N}\sum_{i=1}^{N} m_i, \tag{4}$$

and

$$\overline{m^2} = \frac{1}{N}\sum_{i=1}^{N} m_i^2, \tag{5}$$

f_{MLE} is a nonlinear function that depends on the measurements and their squares (e.g. the right side of the equation). Appendix Figure 1 demonstrates f_{MLE} for N = 2. Evidently, MLE for different combinations of measurements is convex. This indicates a larger values of m_i contribute more to f_{MLE}. This is somewhat counterintuitive because larger m_i is typically associated with larger s whose measurement is less reliable. Nonetheless, in the presence of scalar noise, larger measurements contribute more to f_{MLE}.

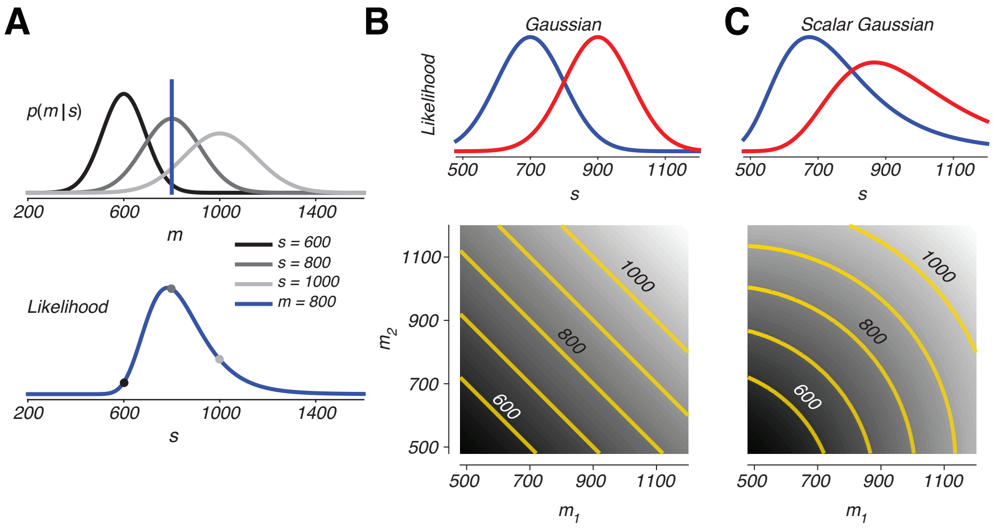

Appendix Figure 1. Impact of scalar variability on magnitude estimation. (A) Origin of the skew in the likelihood function for a scalar Gaussian noise process. Top: the distribution of measurements (m) for a sample of 600, 800, or 1000 with the Weber fraction for measurement, w_m, set to 0.15. Scalar variability results in a Gaussian of increasing variance for increasing sample intervals. Vertical line plots a hypothetical measurement of 800 which could, with differing likelihoods, come from any of the three distributions. Notice that the large value of s is more likely than the small value of s. Bottom: The likelihood of each sample interval for a measurement of 800 (blue). Black, dark gray, and light gray circles plot the relative likelihood that the sample interval was 600, 800, or 1000, respectively. Extrapolation from these examples provides intuition for the long tail (skew) in the likelihood of a scalar Gaussian. (B-C) Consequences of skew in the likelihood function for measurement integration. Top row: likelihood functions for two measurements, m_1 = 700 and m_2 = 900 (blue and red, respectively) for (B) Gaussian and (C) scalar Gaussian likelihood functions. Bottom row: mapping functions for maximum likelihood estimators (MLE) corresponding to each likelihood function. Grayscale indicates the magnitude estimate where whiter points indicate greater magnitudes; contours plot loci of equal estimates for different combinations of measurements for estimates from 600 to 1000, spaced by 100. Measurements are given equal weight under the Gaussian noise model which has no skew. This linearity appears in the mapping function as straight contours in the (m_1, m_2) plane (panel B). Skew in the likelihood function leads to a discounting of the smaller measurement in the MLE, as revealed by the curvature of the contours of the associated mapping function (panel C).
